# Transcription Factor WRKY33 Mediates the Phosphate Deficiency-Induced Remodeling of Root Architecture by Modulating Iron Homeostasis in *Arabidopsis* Roots

**DOI:** 10.3390/ijms22179275

**Published:** 2021-08-27

**Authors:** Nuo Shen, Sifan Hou, Guoqing Tu, Wenzhi Lan, Yanping Jing

**Affiliations:** 1 State Key Laboratory for Pharmaceutical Biotechnology, College of Life Sciences, Nanjing University, Nanjing 210093, China; sn19921112520@163.com (N.S.); nju-sifanhou@163.com (S.H.); 18205098961@163.com (G.T.); 2 College of Life Sciences, Northwest University, Xi’an 710069, China

**Keywords:** phosphate deficiency, root architecture, iron homeostasis, transcription factor

## Abstract

The remodeling of root architecture is regarded as a major development to improve the plant’s adaptivity to phosphate (Pi)-deficient conditions. The WRKY transcription factors family has been reported to regulate the Pi-deficiency-induced systemic responses by affecting Pi absorption or transportation. Whether these transcription factors act as a regulator to mediate the Pi-deficiency-induced remodeling of root architecture, a typical local response, is still unclear. Here, we identified an *Arabidopsis* transcription factor, WRKY33, that acted as a negative regulator to mediate the Pi-deficiency-induced remodeling of root architecture. The disruption of WRKY33 in wrky33-2 mutant increased the plant’s low Pi sensitivity by further inhibiting the primary root growth and promoting the formation of root hair. Furthermore, we revealed that WRKY33 negatively regulated the remodeling of root architecture by controlling the transcriptional expression of ALMT1 under Pi-deficient conditions, which further mediated the Fe^3+^ accumulation in root tips to inhibit the root growth. In conclusion, this study demonstrates a previously unrecognized signaling crosstalk between WRKY33 and the ALMT1-mediated malate transport system to regulate the Pi deficiency responses.

## 1. Introduction

Phosphorus (P) is an essential macronutrient required for constitution of several biomolecules and for regulation of biological and metabolic processes in plants [1]. The phosphorus is acquired by plants in the form of inorganic phosphate (Pi) [2]. In soil, Pi exists at low available concentrations (typically around 1–10 μM in the soil solution) due to its ability to form insoluble complexes with cations, especially with aluminum and iron under acidic conditions and with calcium under alkaline conditions [2,3,4]. Therefore, Pi deficiency is termed as an important abiotic stress that restricts agricultural production [5,6].

Phosphate deficiency triggers a series of Pi starvation responses (PSRs) in plants to reduce the phosphorus usage and increase the phosphorus uptake and recycling [7,8,9,10,11]. The PSRs are divided into two independent signaling pathways, termed the local response and the systemic response [11,12]. The systemic response to Pi deficiency involves increasing the Pi transport through increasing the expression of high-affinity transporters, enhancing the Pi recovery through secretion of phosphatases, and promoting the Pi recycling through catabolism of phospholipids [13,14]. The local response to Pi deficiency is mainly determined by modifying the root system architecture (RSA), including inhibiting the primary root growth and increasing the root hair density and length, which is thought to maximize Pi acquisition in the topsoil [15,16,17,18]. Several key molecular components have been identified to regulate the primary root growth under Pi deficiency. The *Arabidopsis* low phosphate root1 (LPR1) and its close homolog LPR2 belong to the multicopper oxidase family, which positively regulate the Pi-deficiency-induced root growth inhibition by promoting reactive oxygen species (ROS) generation and callose deposition in the root apex zone [19,20]. The zinc-finger transcription factor sensitive to proton rhizotoxicity (STOP1) and malate transporter aluminum-activated malate transporter 1 (ALMT1) have been reported to positively regulate the Pi deficiency responses [21,22]. STOP1 induces the transcriptional expression of ALMT1 under Pi-deficient conditions, which further promotes the excretion of malate into apoplast to aggregate the Fe^3+^, followed by a Fe^2+^ oxidation step to produce ROS and inhibit root growth [20,21,22]. In contrast, The *Arabidopsis PHOSPHATE DEFICIENCY RESPONSE 2* (*PDR2*) encodes an endoplasmic reticulum (ER)-localized P5-type ATPase, which negatively regulates the Pi deficiency-induced root growth inhibition; the mutation of *PDR2* is hypersensitive to Pi deficiency [23]. The aluminum toxicity responses protein ALUMINUM SENSITIVE3 (ALS3) interacts with Sensitive To Aluminum Rhizotoxicity1 (STAR1) to form a putative ATP-binding cassette transporter complex in tonoplasts, which regulates the RSA under Pi deficiency [24,25]. The loss-of-function allele of *ALS3,* also termed as *hypersensitive to Pi starvation10* (*hsp10*), is hypersensitive to Pi deficiency in root growth [26].

Transcription factors (TFs) are required to regulate the various metabolic processes in plants by affecting the gene expression from the transcriptional level. The *WRKY* gene family is the member of TFs that plays critical roles in plant metabolic processes in response to biotic and abiotic stresses [27]. In *Arabidopsis,* the *WRKY* gene family contains 74 members, and the structure is highly conserved, which contains a conserved amino acid sequence motif WRKYGQK at N-termini and a novel zinc-finger-like motif Cys(2)-His(2) or Cys(2)-HisCys at C-termini [28,29]. WRKYs have been reported to regulate the Pi-deficiency-induced systemic response in *Arabidopsis* [30,31,32]. WRKY75 is the first identified member that positively regulates the several Pi-deficiency-induced genes, such as *Mt4/TPS1-like* genes and high-affinity Pi transporters (*PHT1;1* and *PHT1;4*) to respond to low Pi signaling [30]. WRKY6 mediates the Pi deficiency response by negatively regulating the *PHOSPHATE1* (*PHO1*) expression; the proteolysis of WRKY6 from WRKY6-PHO1 complex leads to release of PHO1 and promotes the Pi transportation [31]. In addition, WRKY42 also interacts with WRKY6 to inhibit the *PHO1* expression through directly binding to W-boxes of the *PHO1* promoter [31]. WRKY45 has been reported to regulate the low Pi stress by increasing the *PHOSPHATE TRANSPORTER1;1* (*PHT1;1*) expression and promoting the Pi uptake [32].

The WRKY family plays an important role in regulating the systemic response under Pi deficiency conditions. However, whether these TFs mediate the Pi-deficiency-induced remodeling of root structure is unclear. In this study, we revealed that *Arabidopsis* WRKY33 acted as a negative regulator to mediate the Pi deficiency-induced remodeling of RSA, including to inhibit the primary root growth and to enhance the root hair density and length. We further revealed that WRKY33 regulated the Fe homeostasis to respond to the low Pi signaling. Finally, we found that WRKY33 intersected with the STOP1-ALMT1 component to mediate Fe^3+^ deposition in root tips, which was followed by controlling root growth and root hair development. This study demonstrates a previously unrecognized signaling crosstalk between WRKY33 and the ALMT1-mediated malate transport system to regulate the Pi deficiency responses.

## 2. Results

### 2.1. WRKY33 Regulates the Pi Deficiency Responses in Arabidopsis

**Figure 1:**
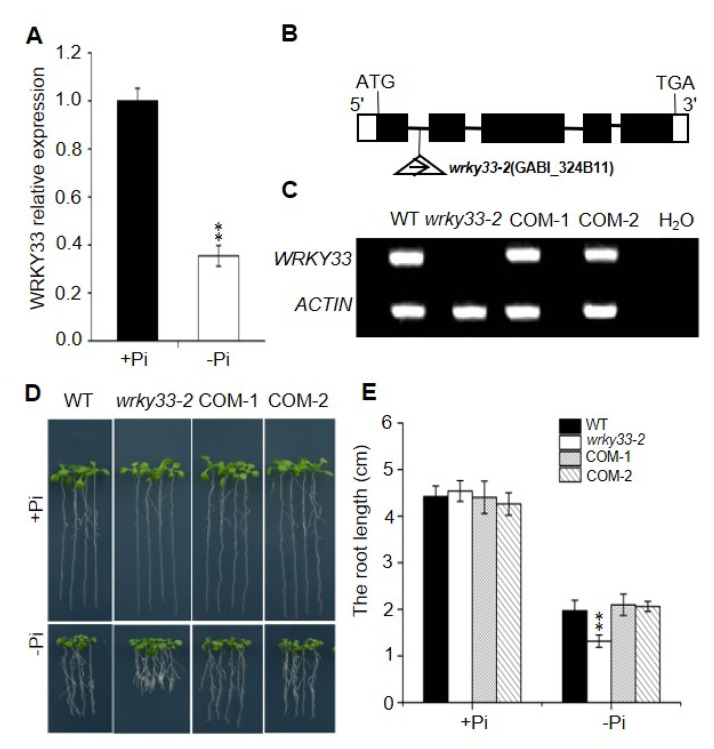
WRKY33 mediated the Pi-deficiency-induced root growth inhibition. (**A**) Quantitative reverse transcription polymerase chain reaction (qRT-PCR) analysis of WRKY33 expression level. Four-day-old wild-type (WT) plants were transferred to Pi-sufficient (+Pi) or Pi-deficienct (−Pi) medium for 2 days. The *WRKY33* expression under Pi-sufficient (+Pi) was set as 1.0, and the Pi-deficiency treatment level was normalized to the Pi-sufficient (+Pi) level. Data are mean ± SD from three replicate experiments. (**B**) Schematic map of T-DNA insertion location of *wrky33-2* mutant. Black boxes, lines, and triangles represent exons, introns, and the position of the T-DNA insertion, respectively. The while boxes indicate the 5′ or 3′ UTRs. (**C**) RT-PCR analysis of the transcriptional level of WRKY33 in WT, wrky33-2, and complementation lines (COM-1, COM-2). Water was used as a negative control. (**D**) Growth phenotypes of WT, wrky33-2, COM-1, and COM-2. Four-day-old seedings were transferred to a Pi-sufficient (+Pi) or a Pi-deficient (−Pi) medium for six days. (**E**) The statistical analysis of the primary root length as indicated in (**D**). Data are means ± SD from three independent experiments (*n* = 15; *n* represents the number of samples). Asterisks in (**A**,**E**) indicate a significant difference from the WT (Tukey’s test; **, *p* < 0.01).

The WRKY33 transcription factor belongs to the WRKY superfamily and plays an important role to regulate the biotic and abiotic stress in *Arabidopsis* [33,34,35]. To investigate whether WRKY33 regulates the Pi deficiency responses in *Arabidopsis*, we examined the transcriptional expression of *WRKY33* in wild-type plants. As shown in Figure 1A, the Pi-deficient condition significantly decreased the *WRKY33* expression level, suggesting that *WRKY33* functions in Pi-deficiency signaling. We continually examined the role of *WRKY33* in response to Pi deficiency by using genetic analysis of the putative T-DNA insertion knockout mutants *wrky33-2* (GABI_324B11) (Figure 1B,C). There were no significant differences in primary root growth between *wrky33-2* and wild-type plants when grown under Pi-sufficient (+Pi) medium (half-strength Murashige and Skoog medium with 625 μM Pi) (Figure 1D). Under Pi-deficient (−Pi) medium (half-strength Murashige and Skoog medium with 10 μM Pi), the root growth in wild type seedlings was inhibited; however, the *wrky33-2* seedlings displayed an aggravated Pi-deficient growth phenotype with shorter roots compared with these in wild-type plants (Figure 1D,E). The time course of Pi-deficient (−Pi) treatment further supported this point (Figure S1). To prove whether Pi homeostasis affects the root growth, we measured the Pi content in the shoots and roots of wild-type and *wrky33-2* plants under Pi-sufficient or Pi-deficient conditions. There were no significant differences in the Pi content of the roots and shoots between wild-type and *wrky33-2* mutant plants under Pi-sufficient or Pi-deficient conditions (Figure S2), suggesting that the difference in Pi-deficiency-induced root growth inhibition between wild-type and *wrky33-2* plants was independent with Pi homeostasis. In addition, we also confirmed that *WRKY33* specifically functioned in Pi-deficiency signaling, as disruption of *WRKY33* did not further change the root growth in other nutrients, including nitrogen, potassium, or Fe-deficient conditions (Figure S3).

To further confirm that the phenotype of the *wrky33-2* mutant resulted from disruption of *WRKY33*, a 3311 bp genomic fragment of the *WRKY33* (containing a 1247 bp promoter and a 2064 bp genomic region from translation initiation codon to termination codon) was transformed into *wrky33-2* for the complementation test. The transcript levels of *WRKY33* in the transformants were measured by real-time quantitative polymerase chain reaction (RT-qPCR) (Figure S4). We selected two representative transgenic lines based on their similar *WRKY33* expression levels with wild-type plants and referred to them as COM-1 and COM-2 (Figure 1C and Figure S4). We found that the primary root growth in COM-1 and COM-2 lines displayed similar growth as the wild type under Pi-deficient medium (Figure 1D,E), indicating that the hypersensitive phenotype of *wrky33-2* mutant to Pi deficiency was due to disruption of *WRKY33*.

**Figure 2:**
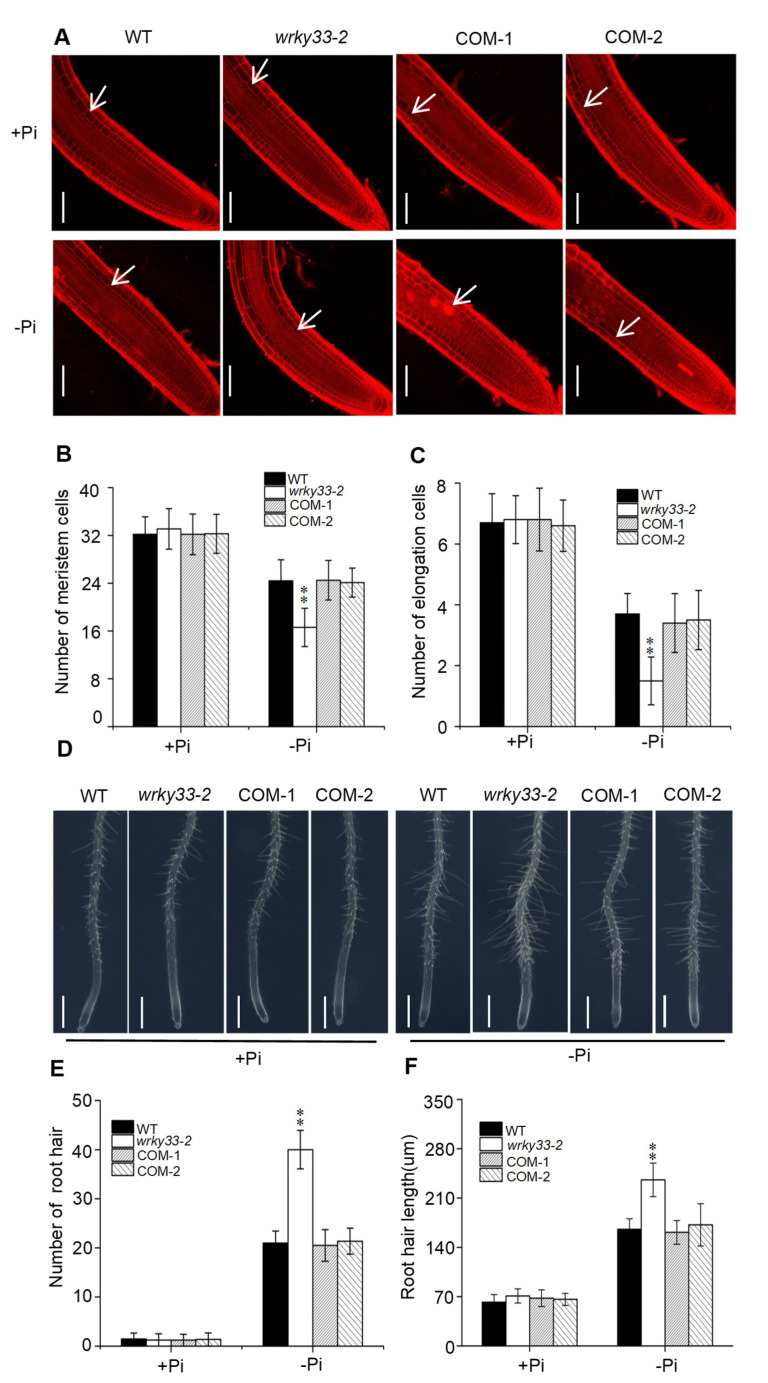
WRKY33 controls the root architecture under the Pi-deficient condition. (**A**) Confocal images of root tips of WT and mutants with propidium iodide staining. Four-day-old seedlings of WT and mutants were transferred to Pi deficiency (−Pi) or Pi sufficiency (+Pi) for three days. White arrows indicate the position above which is the last meristem cell. Bar = 50 μm. Statistics of the number of meristematic cells (**B**) and elongation cells (**C**) in roots as indicated in (**A**). The cortex cell between the quiescent center and the first elongated cell were countered as the meristem cells. Data are means ± SD from three independent experiments (*n* = 8; *n* represents the number of samples). (**D**) Root hair growth phenotype. Four-day-old seedings of WT, *wrky33-2*, COM-1, and COM-2 were transferred to a Pi-sufficient (+Pi) or a Pi-deficient (−Pi) medium for three days. The experiments were repeated three times with similar results. Bar = 200 μm. (**E**,**F**) The root hair number (**E**) and the root hair length (**F**). The hair density and length measured within a 1 mm zone in the root starting from the root tip. Data are means ± SD from three independent experiments (*n* = 15; *n* represents the number of samples). Asterisks in (**B**,**C**,**E**,**F**) indicate a significant difference from the WT (Tukey’s test; **, *p* < 0.01).

The primary root growth inhibition induced by Pi deficiency results from a reduction in root cell elongation and cell division [18]. To analyze the cell status under the Pi-deficient condition, we stained the roots with propidium iodide to analyze the root structure changes. The cell elongation and cell division in the *wrky33-2* mutant did not show significant differences compared with the wild-type, COM-1, and COM-2 plants under Pi-sufficient (+Pi) medium (Figure 2A–C). When the plants were placed under the Pi-deficient condition, the number of meristematic and elongating cells in wild type, COM-1, and COM-2 plants were reduced; the number of these cells in wrky33-2 mutants were further decreased, which is consistent with the root growth under the low Pi condition (Figure 2A–C). In addition, we also observed that Pi deficiency stimulated root hair growth, including increased root hair length and root hair number (Figure 2D–F). The root hair growth in the *wrky33-2* mutant was much stronger than those in other seedlings (Figure 2E,F). Taken together, these results indicate that WRKY33 plays an important role in Pi deficiency-induced remodeling of root structure.

### 2.2. WRKY33 Negatively Regulates the Fe Accumulation in Roots under Pi Deficiency

**Figure 3:**
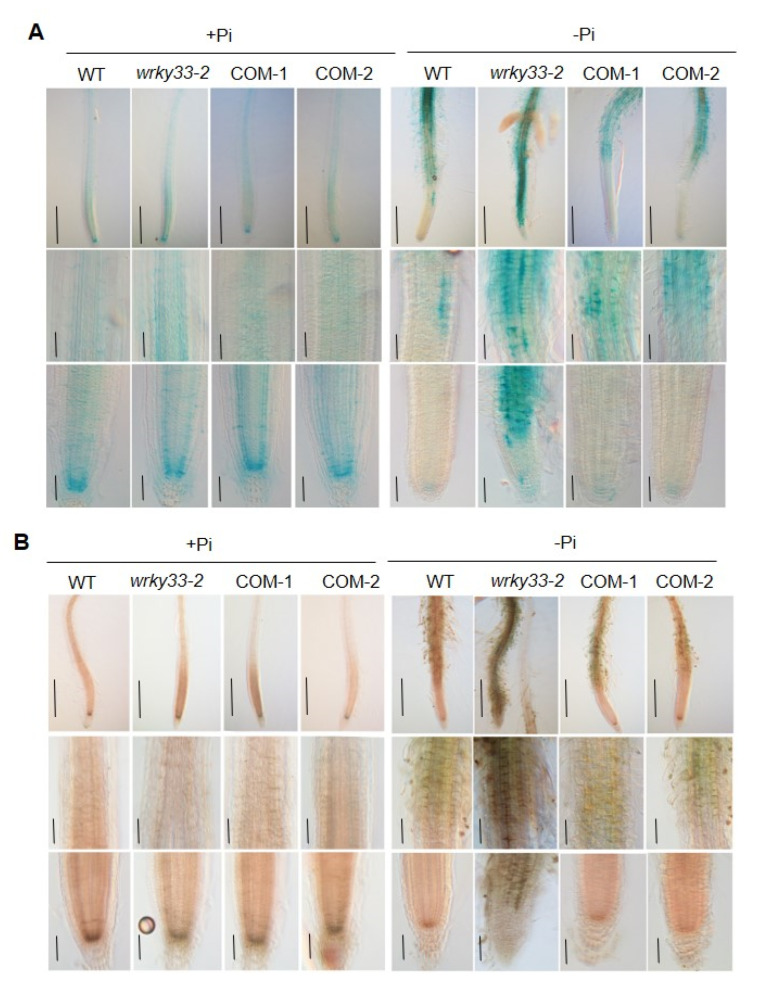
WRKY33 regulates the Fe accumulation under Pi deficiency. (**A**) Fe staining by the Perls method. (**B**) Fe staining by the Perls/DAB method. Four-day-old seedings of WT, *wrky33-2*, COM-1, and COM-2 seedlings were grown on Pi-sufficient (+Pi) or Pi-insufficient (−Pi) for three days; the roots were stained to observe the Fe accumulation. The top, middle, and bottom rows in each section are photographs of the whole root, higher magnification of the maturation zone and the transition zone, and higher magnification of the apical region, including the root cap and meristem, respectively. The experiments were repeated three times with similar results. Bar = 50 μm.

It has been proved that the Pi deficiency promoted the accumulation of Fe in roots [19,20,36]. To investigate whether WRKY33 functions in roots to mediate the Fe accumulation under Pi deficiency, we detected the Fe content in roots by using the Perls staining method, which mainly stains labile (nonheme) Fe^3+^ [37]. Under the Pi-sufficient condition, the Fe^3+^ mainly accumulated in the root stem cell niche (SCN), including the quiescent center and its surrounding initials, as well as in the cortex of the root apex (Figure 3A), which is consistent with the previous result [20]. The Fe^3+^ accumulation in wild-type, *wrky33-2,* COM-1, and COM-2 roots did not show a significant difference (Figure 3A). When the roots were placed under the Pi-deficient medium, the Fe^3+^ accumulation in the SCN and the cortex was dramatically reduced; however, the root maturation zone displayed a strong Fe^3+^ accumulation (Figure 3A). The *wrky33-2* roots showed a stronger Fe^3+^ staining signals than the other three materials (Figure 3A). A similar Fe distribution pattern was observed when a more sensitive Perls/DAB (diaminobenzidine) staining method was used (Figure 3B). In this method, the Perls staining was intensified with the addition of DAB to stain both Fe^2+^ and Fe^3+^ [38]. These results indicate that WRKY33 plays a negative role in the root to mediate the Fe accumulation under Pi-deficient conditions.

### 2.3. WRKY33 Mediates the Plant Pi Deficiency Responses Dependent on Iron Homeostasis

**Figure 4:**
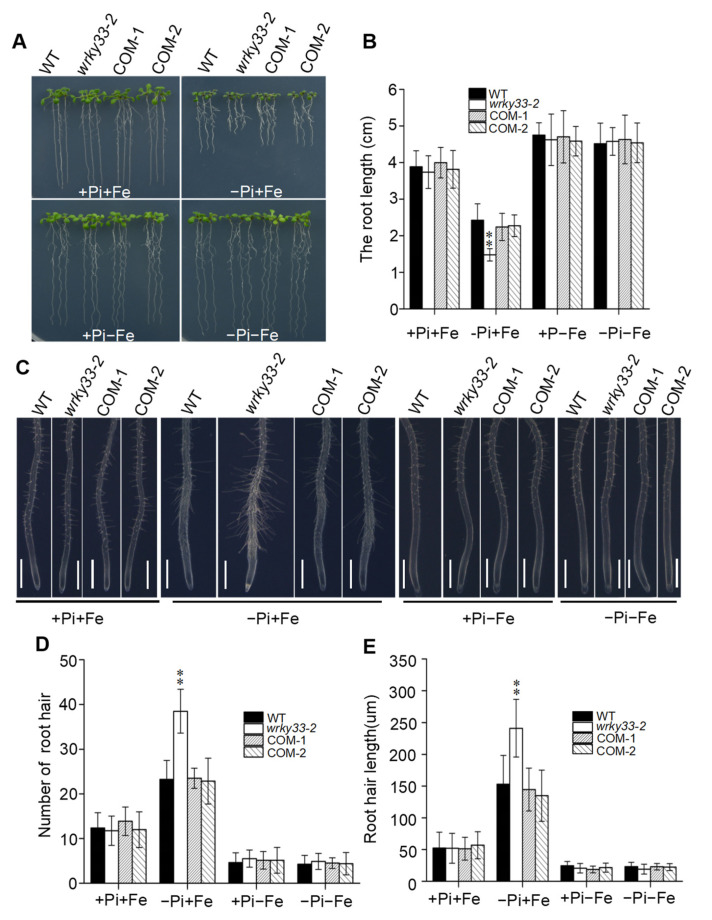
WRKY33 regulates the low Pi signaling that intersects with Fe signaling. (**A**) The growth phenotype of wild-type (WT) and mutant seedlings. Four-day-old seedings were transferred to +Pi +Fe, −Pi+Fe, +Pi−Fe, and −Pi−Fe medium for six days. (**B**) The statistical analysis of the primary root length as indicated in (**A**). Data are means ± SD from three independent experiments (*n* = 15; *n* represents the number of samples). (**C**) Root hair growth in WT, *wrky33-2*, and COM-1 and COM-2 seedlings. Four-day-old seedings were transferred to +Pi+Fe, –Pi+Fe, +Pi−Fe, and −Pi−Fe medium for three days. The experiments were repeated three times with similar results. Bar = 200 μm. (**D**,**E**) Root hair number (**D**) and root hair length (**E**) as indicated in (**C**). The hair density and length measured within a 1 mm zone in the root starting from the root tip. Data are means ± SD from three independent experiments (*n* = 15). Asterisks in (**B**,**D**,**E**) indicate a significant difference from the WT (Tukey’s test; **, *p* < 0.01).

The accumulation of Fe was required to inhibit the root growth under Pi deficiency [19,36]. To further examine whether the intensified inhibition of primary root growth in the *wrky33-2* mutant under the Pi-deficient medium was a result of the overaccumulation of Fe in the root tip, we removed the Fe element from the Pi-sufficient or Pi-deficient medium to observe the primary root growth. As shown in Figure 4A–E, the removing of Fe from the Pi-deficient medium significantly suppressed the low Pi effects in root growth and root hair formation, and the root growth and root hair formation in the *wrky33-2* mutant did not show a significant difference compared with wild-type, COM-1, and COM-2 seedings. Both of four materials did not detect the Fe staining signaling when the root was placed under the −Pi−Fe medium (Figure S5). In addition, we added the ferrozine into the Pi-deficient medium [3-(2-pyridyl)-5,6-bis (4-phenylsulfonic acid)-1,2,4-triazine], a chelator specific for Fe^2+^, and found that ferrozine significantly suppressed the low Pi effects in both WT and *wrky33-2* plants (Figure S6). Taken together, these results imply that the hypersensitivity of *wrky33-2* to low Pi is result of overaccumulation of Fe in roots.

### 2.4. WRKY33 Intersects with ALMT1 to Regulate the Plant Pi Deficiency Responses

**Figure 5:**
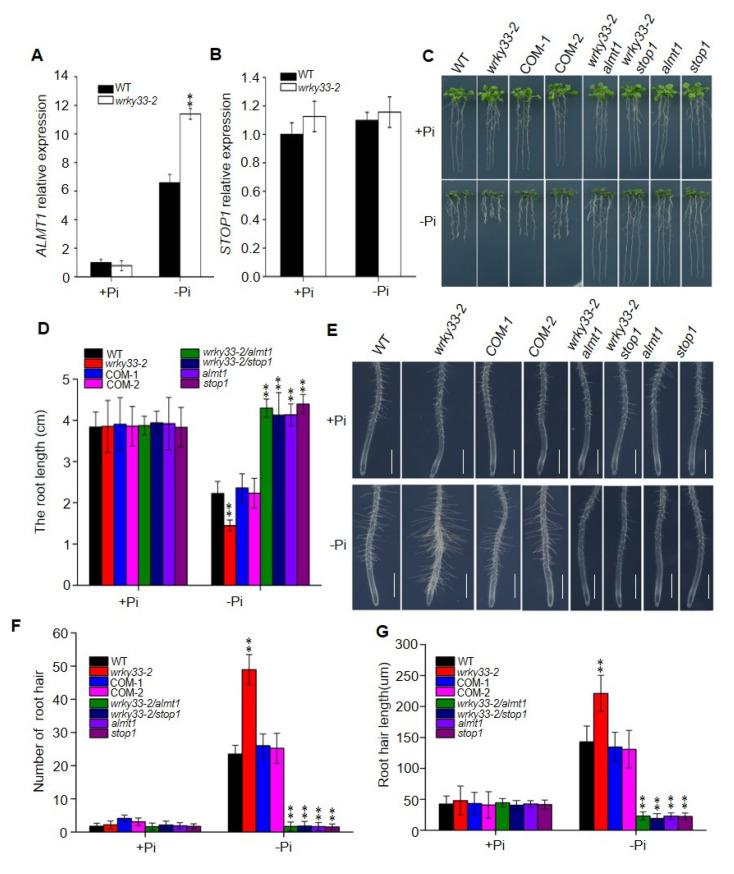
WRKY33 interacts with ALMT1 to regulate Pi deficiency responses. (**A**,**B**) The RT-qPCR assay of *ALMT1* (**A**) and *STOP1* (**B**) expression in wild-type (WT) and *wrky33-2* mutant plants. Four-day-old seedlings were grown on a Pi-sufficient (+Pi) or a Pi-deficient (−Pi) medium for three days. The whole plants were excised for RNA extraction and qRT-PCR analysis. *ACTIN2* was used as the internal reference gene. (**C**) The growth phenotype of WT and mutants on +Pi and −Pi media. Four-day-old seedings were transferred to a Pi-sufficient (+Pi) or a Pi-deficient (−Pi) medium for six days. (**D**) The statistical analysis of the primary root length as indicated in (**C**). Data are means ± SD from three independent experiments (*n* = 15; *n* represents the number of samples). (**E**) Root hair growth in WT, *wrky33-2*, complementation lines, *wrky33-2 almt1*, *wrky332 stop1*, *almt1*, and *stop1* seedlings. Four-day-old seedings were transferred to a Pi-sufficient (+Pi) or a Pi-deficient (−Pi) medium for three days. Bar = 200 μm. (**F**,**G**) The root hair number (**F**) and the root hair length (**G**) as indicated in (**E**). The hair density and length measured within a 1 mm zone in the root starting from the root tip. Data are means ± SD from three independent experiments (*n* = 15; *n* represents the number of samples). Asterisks in (**A**,**B**,**D**,**F**,**G**) indicate a significant difference from the WT (Tukey’s test; **, *p* < 0.01).

In *Arabidopsis*, STOP1 and ALMT1 play important roles in regulating Pi-deficiency-induced root growth inhibition. STOP1 controls the expression of *ALMT1* to mediate the malate’s release into the apoplast and to activate the Fe-redox-cycling system to produce ROS [20,21]. To determine whether WRKY33 intersects with the STOP1-ALMT1 system to regulate the Pi deficiency responses, we first analyzed the mRNA expression levels by using quantitative reverse transcription polymerase chain reaction (qRT-PCR) methods. The expression of *ALMT1* in wild-type roots was significantly induced by low Pi signaling, which is consistent with previous results (Figure 5A) [21]. The Pi deficiency-induced *ALMT1* expression was further increased by disruption of *WRKY33* (Figure 5A). However, the mRNA levels of *STOP1* did not change by altering the Pi content in the medium (Figure 5B); these results are in agreement with the previous reports that STOP1 acted as a sensor but was not transcriptionally induced by Pi deficiency [21].

**Figure 6:**
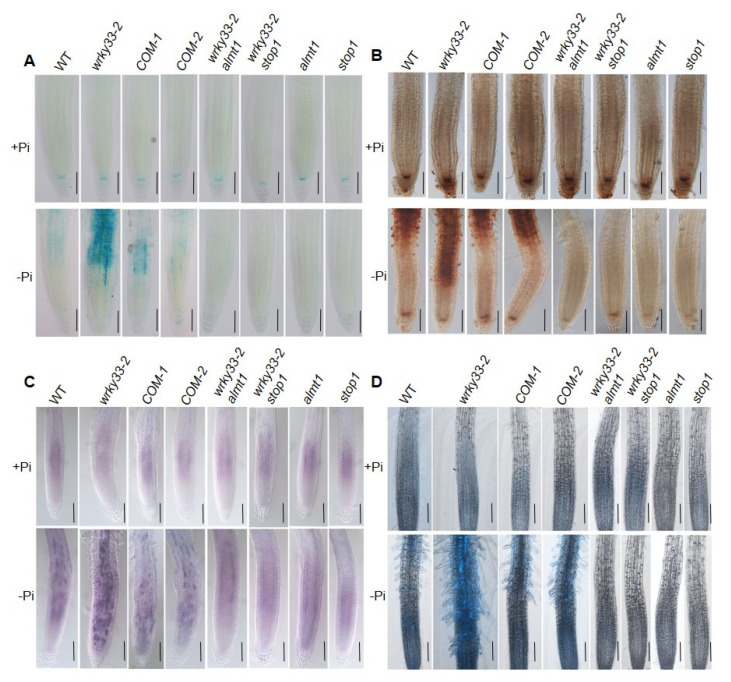
Pi deficiency-induced Fe, ROS, and callose deposition in root apex. (**A**,**B**) Fe staining by Perls (**A**) and Perls/DAB (**B**). Four-day-old plants grown on +Pi or −Pi medium for three days. The experiments were repeated three times with similar results. Bar = 50 um. (**C**) NBT staining of superoxide (O_2_^–^) anion in WT and mutant roots. Four-day-old seedlings were transferred to +Pi or −Pi medium for three days; the roots were stained with 1 mM NBT solution for 60 min and photographed under microscopy. Bar = 50 μm. (**D**) Aniline blue staining of callose in primary roots after transfer of four-day-old plants to +Pi or −Pi medium for three days. Bar = 50 μm.

To confirm the genetic relationship between WRKY33 and the STOP1-ALMT1 system, we continually observed the root growth in *almt1 wrky33-2* and *stop1 wrky33-2* double mutants by hybridizing the *wrky33-2* with *almt1* and *stop1*, respectively (Figure S6). The primary root and root hair growth in *stop1 wrky33-2* and *almt1 wrky33-2* double mutants were similar to those in *stop1* and *almt1* seedlings; both of four materials were absolutely insensitive to low Pi treatment (Figure 5C–G). We further analyzed the Fe accumulation and found that *stop1*, *almt1*, *stop1 wrky33-2*, and *almt1* *wrky33-2* roots could not detect the Fe staining signaling under the Pi-deficiency condition (Figure 6A,B). ALMT1 controls the excretion of malate into the apoplast, which is required to mediate the Pi deficiency responses. We further supplemented 1 mM malate to the Pi-deficient medium and analyzed the root growth. When placed the root under a −Pi+malate medium, where the primary root growth in wild-type, *wrky33-2*, *almt1*, *stop1*, *almt1 wrky33-2*, and *stop1 wrky33-2* mutants were significantly inhibited; however, there were no significant differences in root length between each material. Taken together, these results further indicate that the suppression of the *wrky33-2* hypersensitive primary root growth phenotype in the *almt1 wrky33-2* and *stop1 wrky33-2* double mutants resulted from their reduced ability to excrete malate into the root tips, which is consistent with the function of ALMT1 as a malate transporter (Figure S7).

The accumulation of labile Fe^3+^ in root tips induces a potential Fe redox cycle that helps to generate ROS and promote callose deposition [20]. We further observed the ROS and callose deposition in roots. On the Pi-deficient medium, the ROS and callose deposition in *wrky33-2* roots were much higher than those in wild-type, COM-1, and COM-2 roots (Figure 6C,D). Moreover, the *almt1 wrky33-2* and *stop1 wrky33-2* root exhibited diminished ROS and callose deposition under low Pi conditions, which is consistent with *almt1* and *stop1* mutants (Figure 6C,D). In conclusion, these results indicate that WRKY33 intersects with the ALMT1-mediated malate transport system to mediate the Pi deficiency responses.

## 3. Discussion

Pi is essential for plant growth and development; the lack of phosphorus in the soil threatens plant health. Plants have evolved local and systemic responses to cope with Pi limitations through morphological and physiological modifications [39,40,41,42]. The WRKY transcription factors have been reported to regulate the systemic responses in plants to enhance plant adaptability to low Pi conditions; whether those TFs function in local responses to regulate the Pi-deficiency signaling is unknown. In this study, we revealed that *Arabidopsis* WRKY33 negatively regulates the Pi-deficiency signaling by controlling primary root growth and root hair formation. WRKY33 transcriptionally regulated the ALMT1-mediated malate transport system to mediate the Fe accumulation in root tips under Pi deficiency, which further inhibited the primary root growth and promoted the root hair growth. These findings reveal a previously unrecognized signaling pathway by which WRKY33 transcription factor regulates the plant’s local responses to Pi-deficiency signaling.

In response to external Pi deficiency, local responses are initiated to remodel the root system architecture to enhance Pi absorption [39,40,41,42]. This generally involves a cessation of primary root growth and enhanced root hair development [41]. Although some key components, such as LPR1 ferroxidase, ALS3/STAR1 transporter complex, and STOP1-ALMT1 regulatory modules have been identified to regulate the primary root growth under Pi starvation, the underlying molecular mechanism remains unclear [19,21,24]. In this study, we found that WRKY33 transcription factors negatively regulated the Pi deficiency-induced remodeling of root system architecture, a typical local response (Figure 1 and Figure 2). The disruption of *WRKY33* did not alter the Pi absorption and transportation, as the Pi content in roots and shoots of the *wrky33-2* mutant were similar to those in wild-type seedlings under Pi deficiency (Figure S2). These results imply that WRKY33 acts as a negative regulator in local responses, but not systemic responses, to mediate the Pi deficiency in *Arabidopsis*.

Apoplastic Fe accumulation in the root apex is essential for primary root growth inhibition in response to Pi deficiency. The STOP1-ALMT1 component has been reported to positively regulate the PSRs by initiating a Fe-redox cycle to alter the redox status of the root apical meristem and induce the ROS and callose deposition [20,21,43]. In the current study, we proved that WRKY33 negatively regulated the Pi deficiency-induced exhaustion of the root apical meristem by mediating the Fe accumulation in the apoplast of root apical cells through the ALMT1-mediated malate transportation pathway. Disruption of *WRKY33* further promoted the Fe accumulation in root tips compared with those in wild-type roots under the Pi-deficient condition (Figure 3). At the same time, ROS formation and callose deposition occurred in the root tips of *wrky33-2*. However, the disruption of *STOP1* or *ALMT1* in *stop1*, *almt1*, *almt1wrky33-2*, and *stop1 wrky33-2* mutants absolutely blocked the Pi deficiency effects, the primary root growth, root hair formation, Fe accumulation, and ROS production, and callose deposition in these materials displayed no difference between Pi-deficient and Pi-sufficient conditions (Figure 5 and Figure 6). Furthermore, the reduction in Fe in the medium also inhibited the Pi deficiency-induced root growth inhibition in the *wrky33-2* mutant (Figure 4). The ALMT1-mediated excretion of malate into apoplast plays a key role in regulating the PSRs [21]. In this study, the primary root growth inhibition of all materials (WT, *wrky33-2*, *almt1*, *stop1*, *wrky33 almt1*, and *wrky33 stop1*) were more sensitive to −Pi +malate compared with those in the −Pi condition, indicating that the suppression of the *wrky33-2* hypersensitive root phenotype in *wrky33-2 stop1* and *wrky33-2 almt1* double mutants resulted from their reduced ability to excrete malate into the rhizosphere (Figure S8). STOP1 acts as a sensor to low Pi signaling, which induces the ALMT1 expression to initiate the PSRs, but the transcriptional expression of *STOP1* does not change under low Pi conditions [21]. We found that WRKY33 negatively regulated the transcription of *ALMT1* but not *STOP1* under Pi deficiency (Figure 5A), which further supported our points that WRKY33 acted as a negative regulator in the ALMT1-mediated malate transport system under the low Pi condition. How WRKY33 transcriptionally regulates the *ALMT1* demands a more in-depth examination.

**Figure 7:**
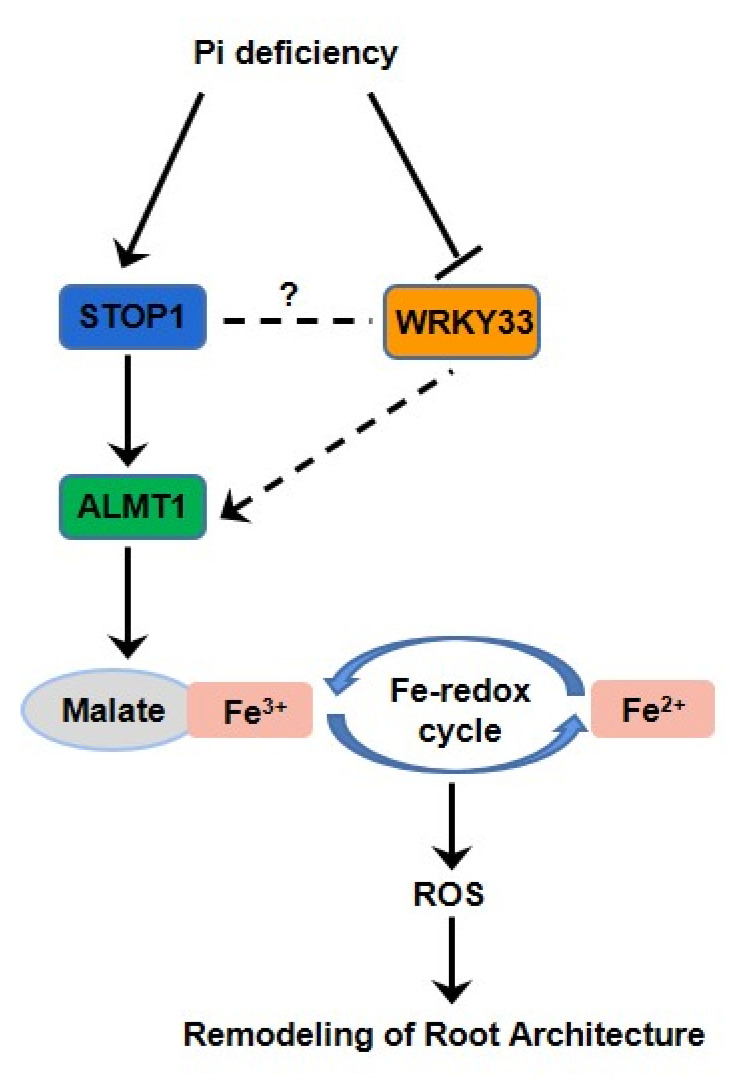
A proposed model of WRKY33’s regulation of the Pi-deficiency responses. Arrows indicate promotion, and perpendicular lines indicate suppression. Broken lines indicate indirect interactions.

In conclusion, the results of this study uncovered a significant role of the WRKY33 in regulating the Pi-deficiency responses by interacting with the ALMT1-mediated malate transport system to control the Fe accumulation in root tip to mediate the Pi deficiency-induced remolding of root system architecture in *Arabidopsis* (Figure 7). It will be interesting to further investigate the potential mechanism of how WRKY33 regulates the ALMT1 component. The research will enrich the PSRs mechanism and increase researcher cognition of how transcription factors regulate the plant’s local responses under Pi-deficient conditions.

## 4. Materials and Methods

### 4.1. Plant Materials and Growth Conditions

Arabidopsis thaliana wild-type (WT; ecotype Columbia-0) was obtained from the Arabidopsis Biological Resource Center. The *wrky33-2* [33], *stop1* [26], and *almt1* [26] mutants were described in previous studies. All *Arabidopsis* lines in this study were Columbia (Col-0) ecotype background. The wrky33 almt1 and wrky33 stop1 homozygous mutants were obtained by hybridization of wrky33 with almt1 and stop1, respectively, following identification by polymerase chain reaction (PCR) using the primers listed in Supplemental Table S1.

For on-plate growth assays, seeds were surface sterilized with 75% (*v*/*v*) ethanol for 5 min and washed in sterilized distilled water, then sown on half-strength Murashige and Skoog (1/2 MS) medium containing 1% (*w*/*v*) sucrose (Aldrich-Sigma, St. Louis, MI, USA) and 1% (*w*/*v*) agar (Sigma-Aldrich, St. Louis, MI, USA). After the seeds were ventilated at 4 °C in the dark for 2 days, the plates were placed vertically in a plant incubator at 22 °C with a 16 h:8 h, light:dark photoperiod (Philips, Amsterdam, Netherlands). The light intensity was 90 µmol/m^2^/s. A half-strength MS medium with 1% (*w*/*v*) sucrose and 1% (*w*/*v*) agar was termed as the standard Pi-sufficient (+Pi) medium, and 10µM KH_2_PO_4_ was used as the Pi resources for Pi-deficiency treatment

### 4.2. RT-PCR and qRT-PCR

The total RNA was isolated from the *Arabidopsis* seedlings using Trizol (Invitrogen, Carlsbad, CA, USA) according to the manufacturer’s instruction. The first-strand cDNA was synthesized by M-MLV Reverse Transcriptase (Promega, Madison, WI, USA). RT-PCR was performed using cDNA for PCR amplification to verify the absence of transcript in mutants with gene-specific primers (Supplemental Table S1). qRT-PCR analysis was carried out using FastStart Universal SYBR Green MasterMix (Roche Diagnostics, Hong Kong) on a CFX Connect Real Time System (Bio-Rad, Berkeley, CA, USA). *ACTIN2* (AT3G18780) was used as the internal standard. Each experiment was repeated three times with three technical replicates, and similar results were obtained. The primers used for qPCR are listed in Table S1.

### 4.3. Pi Content Measurements

Six-day-old wild-type and mutant seedlings were transferred to the Pi-sufficient and Pi-deficient medium for another 6 days. The roots and shoots were collected for Pi content measurement according to the ascorbate-molybdate-antimony method [44].

### 4.4. Perls and Perls/DAB Staining Assays

Perls staining was carried out as described by Roschzttardtz et al. (2009) with minor modifications. In brief, four-day-old seedlings were transferred onto a Pi-sufficient and a Pi-deficient medium for 3 days. For Fe detection with Perls staining, the seedlings were submerged in the staining solution (4% (*v*/*v*) HCl, 4% (*w*/*v*) K-ferrocyanide). After 30 min, the samples were washed with sterile distilled water three times, and the roots were observed under a confocal microscope (BX53, Olympus, Tokyo, Japan).

For Fe detection, Perls/DAB staining was performed according to Balzergue et al. (2017). The final staining concentration of DAB (Sangon Biotech, Shanghai, China) solution was 0.025%. The staining roots were stored in a 0.1 M Na-Pi buffer (pH 7.4) before being photographed. Roots were incubated in chloral hydrate solution (4 g chloral hydrate, 1 mL glycerol, and 2 ml H_2_O) for 1 min and photographed by a confocal microscope (BX53, Olympus, Tokyo, Japan). For each genotype, 10 roots were stained. The experiments were repeated three times.

### 4.5. Root Hairs Assay

To analyze the root hair density and length, four-day-old seedlings grown on half-strength MS agar medium were transferred onto a Pi-sufficient and a Pi-deficienct medium for 3 days. Images were captured using a SZX16 microscope (Olympus, Tokyo, Japan). Image J Software (Version 1.48v, National Institutes of Health, Bethesda, MD, USA) was used to measure root hair density and length within a 1 mm zone in the root starting from the root tip [45].

### 4.6. Plasmid Construction and Plant Transformation

For construction of the genetic complementation vector, the full-length genomic DNA of the WRKY33 fragment (a 3311 bp fragment containing a 1247 bp promoter and a 2064 bp genomic region from translation initiation codon to termination codon) was amplified from the genomic DNA of wild-type seedlings and cloned into the binary vector pCAMBIA-1300 via the PstI-BamHI site. Then, the recombinant plasmid was transformed into the *Agrobacterium tumefaciens* strain GV3101. The complementation lines were generated by the flora dip method [46]. Finally, the transformants were screened with 25 mg/l of hygromycin. The primers used to produce the constructs are listed in Supplemental Table S1.

### 4.7. Confocal Laser-Scanning Microscopy and Staining Procedures

Confocal microscopy was done on a Zeiss, LSM-710 (Oberkochen, Germany). For PI staining, seedlings were directly stained with 5 μM PI (Sigma-Aldrich, St. Louis, MI, USA) and washed 3 times. Callose was stained with 0.1% (*w*/*v*) aniline blue (Sangon Biotech, Shanghai, China) for 1.5 h in a 100 mM na-phosphate buffer (pH 7.2). For NBT staining, the roots were stained with a 1 mM NBT solution (Sangon Biotech, Shanghai, China) for 60 min and photographed under microscopy.

### 4.8. Statistical Analysis

For all experiments, three independent repetitions were performed. Data were subjected to statistical analyses using one-way ANOVA followed by Tukey’s test. Asterisks in the figures denote significant differences as follows: * *p* < 0.05 and ** *p* < 0.01.

## Data Availability

All data supporting the results of the study have been presented as figures.

## Supplementary Materials

The following are available online at https://www.mdpi.com/article/10.3390/ijms22179275/s1.
