# Supplementary material for: Transcription Factor WRKY33 Mediates the Phosphate Deficiency-Induced Remodeling of Root Architecture by Modulating Iron Homeostasis in Arabidopsis Roots
Source: Int J Mol Sci. 2021 Aug 27;22(17):9275. doi: 10.3390/ijms22179275 (PMC8431420; doi:10.3390/ijms22179275)
Supplement: Supplementary file 1 [file ijms-22-09275-s001.zip › ijms-1328697-supplementary.pdf]

International Journal of *Molecular Sciences*

MDPI

**Supplementary datas**

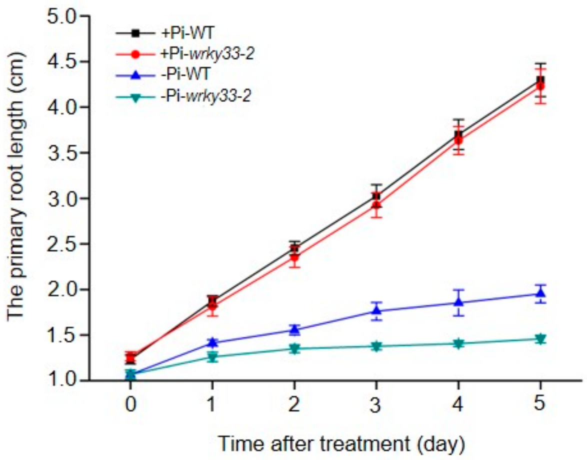


**Supplementary figure S1**. Root length of WT and *wrky33-2* grown on +Pi or −Pi medium for 5 days. Seedings were geminated on 1/2 MS agar medium and 4-day-old seedings were transferred to +Pi or −Pi medium for another 5 days. Data are means ± SD from three independent experiments (n = 8, n represents the number of samples).

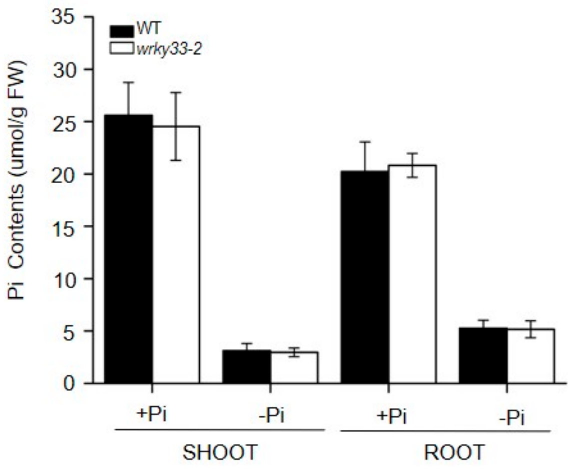


**Supplementary figure S2**. Pi content in roots and shoot of WT and *wrky33-2* mutant. Six-day-old seedlings grown on +Pi or −Pi medium for another 6 days. Roots and shoots were harvested separately for Pi content determination. Data are means ± SD of three replicate experiments.

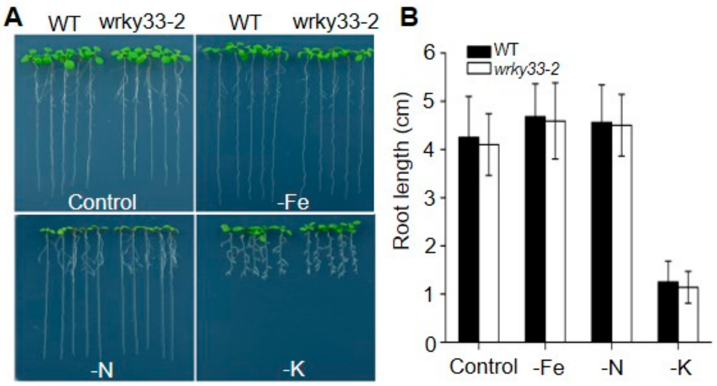


**Supplementary figure S3**. The seedlings growth under nitrogen(N), potassium(K), and Iron (Fe) deficient conditions. (A)The growth phenotypes of WT and *wrky33-2* mutants. Four-day-old seedings were transferred to –N, –K, or –Fe medium for 6 days. (B) The statistical analysis of the primary root length as indicated in (A). Data are means ± SD from three independent experiments (n=15, n represents the number of samples).

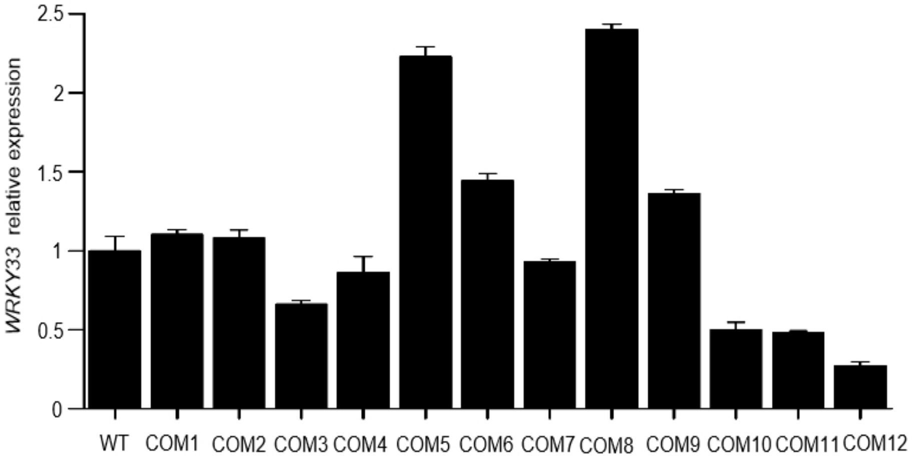


**Supplementary figure S4**. qRT-PCR analysis of *WRKY33* expression in transgenic plants. 7-day-old plants were excised for RNA extraction and qRT-PCR analysis. *ACTIN2* was used as an internal standard. Data are mean ± SD from three independent experiments.

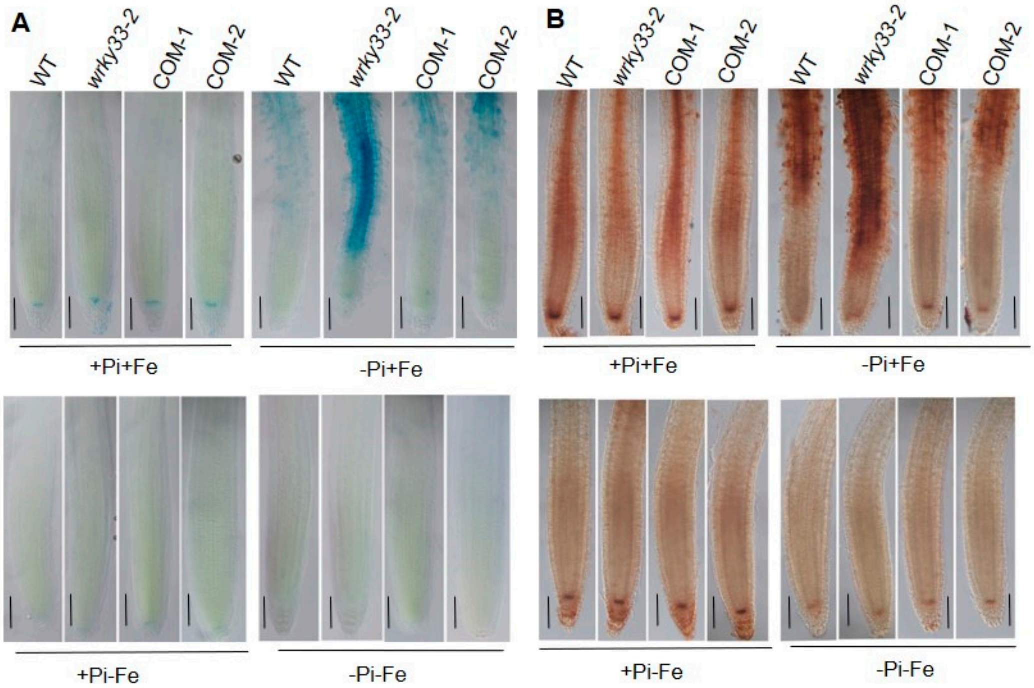


**Supplementary figure S5**. Pi deficiency-induced Fe accumulation in root tips depended on the presence of Fe in the medium. (A-B) Fe staining by Perls (A) and Perls/DAB (B). Four-day-old seedings of WT, *wrky33-2* and complementation lines grown on +Pi+Fe,–Pi+Fe,+Pi–Fe and –Pi–Fe medium for 3 days. Bar=50um.

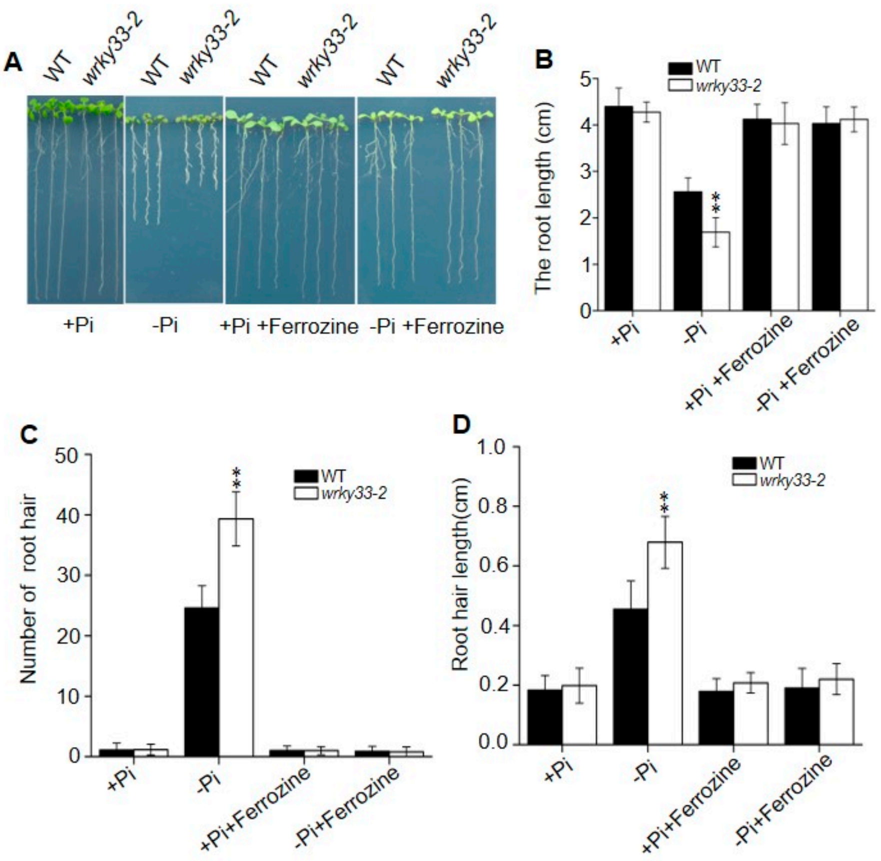


**Supplementary figure S6.** Ferrozine alleviates Pi deficiency-induced root growth inhibition. (**A**) 4-day-old seedings of WT and *wrky33-2* were transferred to +Pi and –Pi medium supplemented with 200 uM ferrozine for 6 days. (**B**) The statistical analysis of the primary root length in (**A**). Data are means ± SD from 3 independent experiments (n = 15). The root hair number (**C**) and root hair length (**D**). Four-day-old seedings of WT and *wrky33-2* grown on +Pi and –Pi medium supplemented with 200 uM ferrozine for 3 days. Data are means ±SD from three independent experiments (n = 15, n represents the number of samples). Asterisks in (**B**), (**C**) and (**D**) indicate a significant difference from the WT (Tukey's test; **, P <0.01).

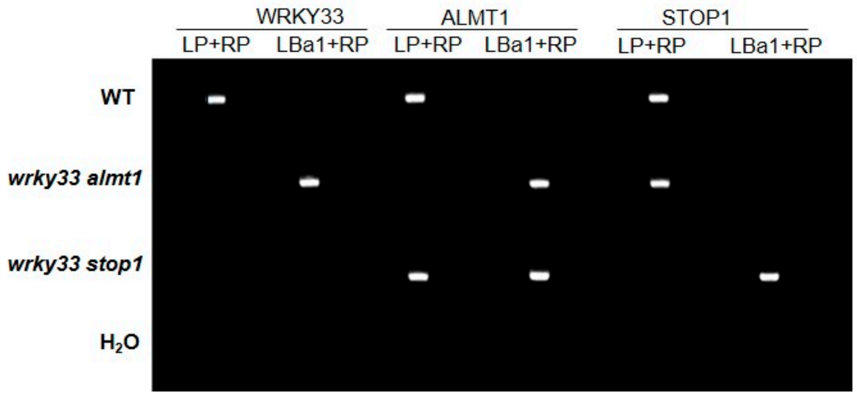


**Supplementary figure S7.** The identification of *wrky33 almt1* and *wrky33 stop1* double mutant. Confirmation of the T-DNA insertion in the *wrky33 almt1* and *wrky33 stop1* double mutant by PCR.

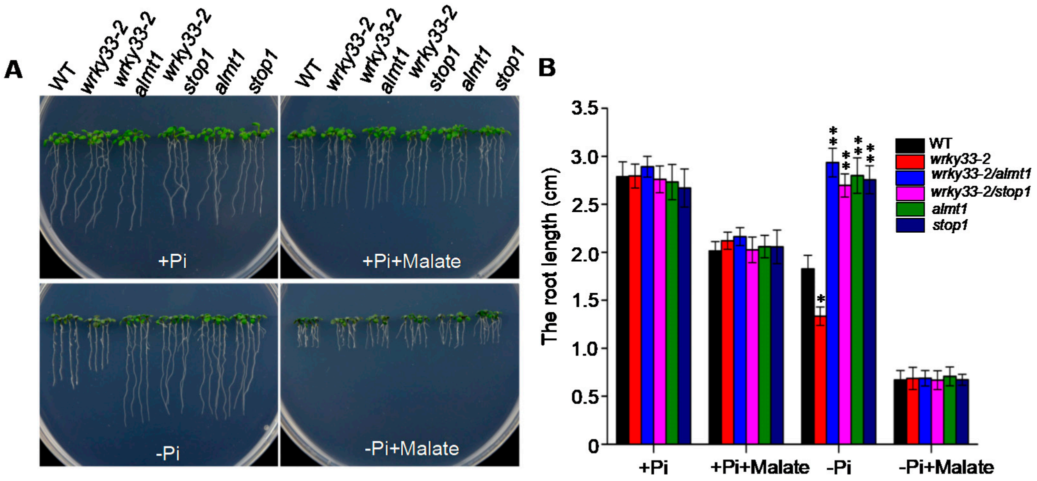


**Supplementary figure S8.** Effect of malate on the root growth under Pi deficiency condition. (A) The growth phenotype of WT, *wrky33-2*, *wrky33-2 almt1*, *wrky332 stop1*, *almt1* and *stop1* seedlings. Four-day-old seedings were transferred to +Pi or -Pi medium supplemented with or without 1 mM malate for 6 days. (B) The statistical analysis of the primary root length in (A). Data are means ±SD from three independent experiments (n = 15, n represents the number of samples). Asterisks in (B) indicate a significant difference from the WT (Tukey's test; *, $P < 0.05$; **, $P < 0.01$).

**Supplementary Table S1**

Primers used in this study

| Primer name | Primer sequences (5'-3') |
|---|---|
| (1) qRT-PCR analyze | |
| *Actin2*-F | CTGTTCTCTCCTTGTACGCCAGT |
| *Actin2*-R | CGGGTAATTCATAGTTCTTCTCGAT |
| *WRKY33*-F | CCATCGGTTGTCCAGTGAGG |
| *WRKY33*-R | GCTCTGTTTGTGGCGTAACC |
| *ALMT1*-F | ACTTGAGAGAGCTGAGTGACC |
| *ALMT1*-R | TCTTCTCGGGTCTTCATTCCC |
| *STOP1*-F | CCAAGTTCCATCTCAAGCTTTTCT |
| *STOP1*-R | TGGGACGTAAAACCTGCGAA |
| **(2) Construction vector** | |
| *WRKY33*-COM-F | AACTGCAGGCCAAAGGGTGTTGTTATTGA |
| *WRKY33*-COM-R | CGGGATCCTCAGGGCATAAACGAATCGA |
| **(4) Identification of mutants** | |
| LBa1 | TGGTTCACGTAGTGGGCCATCG |
| GABI | GGGCTACACTGAATTGCTAGCTC |
| *wrky33-2*-LP | ATGTCAGGTC TCGAAGATAT |
| *wrky33-2*-RP | CTACACAGTG TAGTGATGTC |
| *almt1*-LP | AGTGAGAGAA GGGATTAGAGTAGG |
| *almt1*-RP | GGCAACATGA TGACATGAGTC |
| *stop1*-LP | AAGACGATTT GTGCAACACC |
| *stop1*-RP | CAGACTCACC AACATTCCTG |
